# Quality assurance for point-of-care diagnostic testing: It is not negotiable

**DOI:** 10.4102/ajlm.v5i2.554

**Published:** 2016-10-17

**Authors:** Maurine M. Murtagh

**Affiliations:** 1 International Diagnostics Centre, London School of Hygiene & Tropical Medicine, London, United Kingdom

This special issue of the African Journal of Laboratory Medicine (AJLM) is dedicated to quality assurance for *in vitro* diagnostics (IVDs), which encompasses all aspects of the quality management of diagnostic testing, including quality control of the testing process as well as monitoring and control of the outcomes of that process, which are designed to ensure that test results are as accurate as possible. External quality assessment is an important element of this system and is the focus of most of the articles in this supplement.

Inaccurate IVD testing can have significant adverse impacts for patients. For an individual patient, it means not only a delay in receiving the correct diagnosis, but can also result in improper or unnecessary treatment, which may lead to long-term complications, additional clinic visits and additional cost. For public health programmes, failure to identify pathogens correctly in the event of an outbreak can lead, among other things, to a delay in determining the true cause and extent of an outbreak, and inadequate or incorrect control measures being put into place. In general, poor diagnostic quality leads to poor patient outcomes, increasing morbidity and mortality, and poses a barrier to effective public health interventions. Although it should be self-evident that IVD testing is only valuable if it is accurate, quality assurance has often lagged behind scale-up of testing in resource-limited settings.

With the high prevalence of disease in resource-limited settings, including HIV, malaria and tuberculosis, as well as unexpected outbreaks of infections such as Ebola, dengue and Zika, testing is critical and countries are often exhorted to scale-up testing quickly by the World Health Organization and other national and international stakeholders. However, the health systems in which testing must occur are often weak, fragmented and ill-prepared for any additional testing.

To date, most testing in these countries, especially device-based testing, has taken place in centralised laboratories, while the majority of patients live, and present for treatment, in rural or peri-urban areas far from those facilities. Therefore, effective scale-up of IVDs requires not only increasing capacity for testing centrally, but also decentralising testing to take diagnostics closer to the point of care (POC), with measures to ensure the quality of testing, wherever it is performed.

This has proven to be a difficult task. Using HIV as an example, in addition to well-established laboratory-based platforms for CD4 testing, countries have had access to POC testing platforms for CD4 for more than five years, and many such platforms have been implemented. However, managing a network of POC sites has proven to be challenging, with significant operator and instrument errors, instrument breakdowns, reagent stock-outs and other difficulties plaguing the system.

As countries now try to scale-up early infant diagnosis and viral load testing for HIV to reach the UNAIDS 90-90-90 goals by 2020, it is expected that POC testing will play a significant role, and quality data will provide measurements for impact and success. As illustrated by the articles in this special issue of AJLM, countries are using lessons learned from their experiences with CD4 POC introduction to develop policies and strategic plans for early infant diagnosis and viral load scale-up in which quality assurance, including external quality assessment schemes, are embedded for both laboratory and POC testing. A pilot study in Zimbabwe, for example, demonstrated the importance of using connectivity solutions to facilitate automated and timely reporting of external quality assessment results to monitor instrument and operator performance and to enable corrective actions to be taken as an integral part of a quality assurance system.

Quality assurance is often viewed as an added cost of an IVD system. This is particularly evident in resource-limited settings, where countries must make difficult decisions each year with respect to how to apportion budgets for diagnostics. The London School of Hygiene & Tropical Medicine has developed a simple costing model that can quantify the significant value of diagnostic quality assurance and the serious consequences of not incorporating quality assurance into diagnostic programmes, both in terms of the cost of lives lost and the cost of unnecessary or incorrect treatment.

The consequences of introduction of new diagnostics without quality assurance are dire in terms of lives and disability life years lost. The time has come for all stakeholders in global health to recognise that quality assurance for diagnostic tests and testing are not negotiable. Investments in diagnostic quality assurance systems will pay dividends for years to come, improving both patient outcomes and healthcare economics in-country.

